# Potential of electrospun cationic BSA fibers to guide osteogenic MSC differentiation via surface charge and fibrous topography

**DOI:** 10.1038/s41598-019-56508-6

**Published:** 2019-12-27

**Authors:** Annamarija Raic, Frank Friedrich, Domenic Kratzer, Karen Bieback, Joerg Lahann, Cornelia Lee-Thedieck

**Affiliations:** 1 Leibniz University Hannover, Institute of Cell Biology and Biophysics, Hannover, 30419 Germany; 2 Karlsruhe Institute of Technology (KIT), Institute of Functional Interfaces, Eggenstein-Leopoldshafen, 76344 Germany; 3 Karlsruhe Institute of Technology (KIT), Competence Center for Material Moisture, Eggenstein-Leopoldshafen, 76344 Germany; 4 Institute of Transfusion Medicine and Immunology, Medical Faculty Mannheim, Heidelberg University; German Red Cross Blood Service Baden-Württemberg – Hessen, Mannheim, 68167 Germany; 5 Biointerfaces Institute and Departments of Chemical Engineering, Materials Science and Engineering, Macromolecular Science and Engineering and Biomedical Engineering, University of Michigan, Ann Arbor, MI 48109 USA

**Keywords:** Extracellular matrix, Mesenchymal stem cells, Biomaterials - cells

## Abstract

Large or complex bone fractures often need clinical treatments for sufficient bone repair. New treatment strategies have pursued the idea of using mesenchymal stromal cells (MSCs) in combination with osteoinductive materials to guide differentiation of MSCs into bone cells ensuring complete bone regeneration. To overcome the challenge of developing such materials, fundamental studies are needed to analyze and understand the MSC behavior on modified surfaces of applicable materials for bone healing. For this purpose, we developed a fibrous scaffold resembling the bone/bone marrow extracellular matrix (ECM) based on protein without addition of synthetic polymers. With this biomimetic *in vitro* model we identified the fibrous structure as well as the charge of the material to be responsible for its effects on MSC differentiation. Positive charge was introduced via cationization that additionally supported the stability of the scaffold in cell culture, and acted as nucleation point for mineralization during osteogenesis. Furthermore, we revealed enhanced focal adhesion formation and osteogenic differentiation of MSCs cultured on positively charged protein fibers. This pure protein-based and chemically modifiable, fibrous ECM model allows the investigation of MSC behavior on biomimetic materials to unfold new vistas how to direct cells’ differentiation for the development of new bone regenerating strategies.

## Introduction

After bone fractures mesenchymal stromal cells (MSCs) take on a key role in the *in vivo* bone repair process^1^. They are one prominent cell population in the human bone marrow and apart from accomplishing complex processes, such as bone formation and regeneration, they are also involved in blood hemostasis^2,3^. MSCs have the ability to differentiate into adipocytes and chondrocytes forming fat tissue and cartilage as well as into bone cells – called osteocytes. These bone cells as well as their precursor cells, the osteoblasts are involved in the constant turnover of bone tissue formation^4^. Osteoblasts dissipate calcium and phosphate ions into the subjacent collagenous bone for the formation of hydroxyapatite (Ca_5_(PO_4_)_3_(OH)) for bone stiffening^5,6^. Hence, osteoblasts ensure bone regeneration and bone assembly, the so called ossification, after bone fracture^1^.

In aging western societies, patients with bone injuries are admitted more and more frequently to clinics. Large bone defects can often not sufficiently be repaired by the body itself and require new clinical therapies^1^. The use of donor derived MSCs for triggering the bone regeneration *in vivo* is a promising tool for assisting the healing process. Nevertheless, MSCs can lose their differentiation potential after expansion *in vitro* and transplantation into the site of injury^7^. Therefore, it is important to understand how MSC differentiation into bone cells is controlled and how this process can be guided or improved by externally added factors. Addressing this issue can help to develop e.g. osteoinductive implants. In an ideal scenario, the implant surface will lead to osteogenic differentiation and hence rapid healing of bone defects^8^. In order to cope with this task, *in vitro* systems are required which allow studies regarding cell-material interactions to unfold the impact of material surface properties on MSCs.

*In vitro* systems are often a combination of mimicking the tissue properties and modifying the biomaterial surfaces by coupling of specific factors. Here, soluble factors, known to trigger osteogenic differentiation, are often used^1,4^. Interaction of MSCs with bone morphogenic proteins (BMPs) in an alginate scaffold mimicking the extracellular matrix (ECM) *in vivo* enhances their differentiation into bone cells^9^. Furthermore, modification of *in vitro* model surfaces with the bone substrate hydroxyapatite also shows a positive effect on MSC behavior regarding their osteogenic differentiation^10^. Comparable to tissue, scaffolds can be generated with variation of the rigidity^11^. In *in vitro* experiments, it has been shown that MSCs respond to the substrate stiffness of their surroundings. Stiff growth surfaces support the differentiation to the osteogenic lineage^11^. Furthermore, the topography of substrates such as 2 μm ridges structures revealed an enhanced MSC differentiation towards osteoblasts and surface roughness’s varying from the sub-micron to the micrometer range (∼0.5–4.7 μm) effected the osteogenic commitment and gene expression^12,13^. Several studies reported the effect of a fibrous topography ‒ mimicking the fibrous structure of the ECM ‒ on MSC behavior regarding osteogenic lineage formation^14–16^. Osteogenic differentiation of cells can be enhanced by fiber meshes (randomly oriented) in contrast to aligned fibers which were generated by using the electrospinning technique^16^. In contrast to other scaffolds, in case of fiber meshes, crucial scaffold parameters such as pore size which are known to have an impact on MSC behavior, can be controlled. Fiber meshes with a fiber spacing gradient from 500 μm to 1100 μm improved the osteogenic differentiation of MSCs in culture^17^. These fibers can be spun from synthetic polymers such as Poly(L-lactic acid), Poly(lactide-co-glycolide) and Polycaprolactone with the advantage of biocompatibility, good mechanical properties and stability (no degradation)^18,19^. Furthermore, charged Polyvinylidine fluoride (PVDF) fibers showed an impact on cell shape and collagen mineralization^20^. Nevertheless, incorporation of natural ECM components such as collagen promoted early adhesion and osteogenic gene expression of MSCs in contrast to pure polymer fibers^15,21^. Therefore, pure fibers from natural materials from ECM components are a promising substrate for osteogenic differentiation of MSCs. However, these materials are in most of the cases expensive, often characterized by a fast degradation and have poor mechanical properties^19,22^. Conjugation of aspartic acid (ASP) with Poly(lactic-co-glycolic acid) fibers showed to be an good alternative for protein fibers and revealed a positive effect on expression of key osteogenic markers^23^. Therefore, charged fibers seem to be a promising *in vitro* system for studying MSCs interaction.

We developed a biomimetic *in vitro* system based on an easily available and cost-effective protein – bovine serum albumin (BSA) - which allows observing specific cell-response of MSCs to modified surfaces. In contrast to other fibrous scaffolds, the protein used here as a biopolymer was modified with amino groups to resemble both chemical composition and the fibrous architecture of the ECM in bone tissue. The introduced primary amines are protonated at physiological pH and thus confer positive charge on the protein. Therefore, the synthesized polymer was named as cationized BSA – short cBSA. MSCs were cultured on this synthetic ECM and their cell behavior such as cell morphology and differentiation were investigated. Our present study highlights the importance of the fibrous structure, the amination of BSA – leading to a positively charged cell culture substrate – and the calcifiability of the developed biomaterial for its effects on the osteogenic differentiation of MSCs.

## Results

### Developed protein fibers differ in size and degradation stability

**Figure 1:**
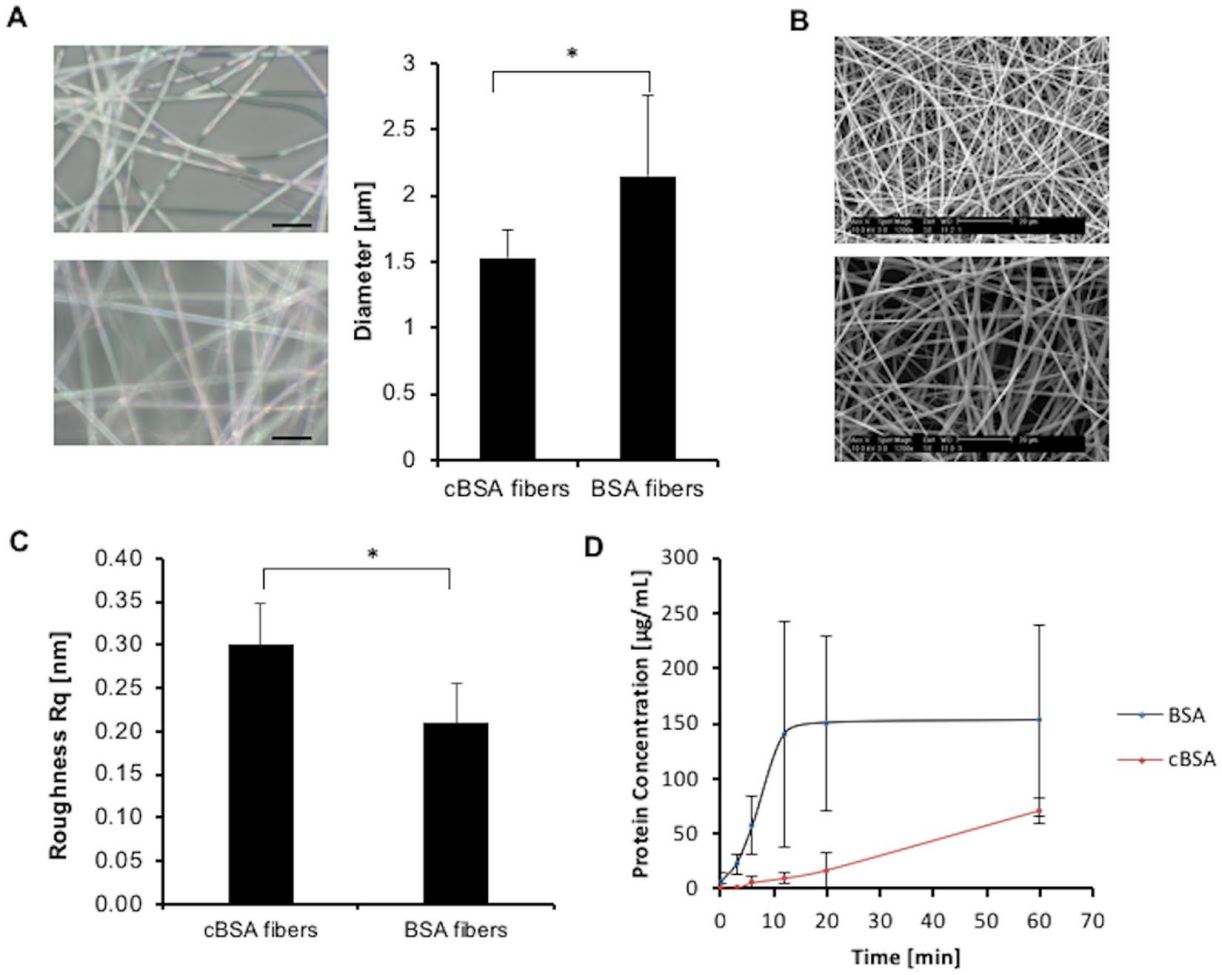
Characterization of cBSA- and BSA-fibers. (**A**) Digital microscopic images of wet cBSA- (above) and BSA-fibers (below) for determination of the fiber diameter (bar graph, right). Scale bar: 10 µm. Bar graph represents the fiber diameter with SD. N = 3 independent experiments. Statistical test: Student’s t-test. (**B**) SEM images of a dense fiber meshwork from cBSA- (above) and BSA-fibers (below) in dried state. Scale bar: 20 µm. (**C**) Bar graph represents the mean surface roughness (Rq) with SD of the different fiber types. Per fiber type 3 different fibers were measured at 3 different positions. Statistical test: Student’s t-test. (**D**) Curve progression of the protein concentration in the supernatant of trypsinized cBSA- (red) and BSA-fibers (black) against the time. Error bars represent the SD. Statistical test: Student’s t-test. Number of independent experiments N = 3; 2 measurements per experiment.

BSA was cationized by coupling ethylenediamine to the carboxyl groups of the protein. The mass of the BSA and cBSA was analyzed by MALDI-TOF mass spectrometry (Fig. S1). Upon functionalization of BSA with ethylenediamine an increase of the molar mass from M_n_ ≈ 67000 Da to M_n_ ≈ 72000 Da was observed. This equals the calculated molar mass of the molecule, when all carboxyl groups were functionalized with ethylenediamine. The developed cBSA-fibers showed a diameter of 1.53 ± 0.22 µm (±SD) whereas the BSA-fibers had a diameter of 2.15 ± 0.61 µm (Fig. 1A). The fibers were spun for 30 min to obtain multilayered fiber mesh (Fig. 1B). Atomic force microscopy (AFM) was utilized in order to obtain information on the surface nanotopography of the applied fiber-types in terms of surface roughness (Rq value). The measurements revealed a mean surface roughness of 0.299 ± 0.048 nm (±SD) for cBSA-fibers and 0.209 ± 0.045 (±SD) nm for BSA-fibers showing that both fiber types possess a smooth surface structure (Fig. 1C, Table 1, detailed information on the AFM measurements can be found in the supplementary information, Fig. S2). Nevertheless, although the Rq values for both fiber types were small indicating smooth surfaces, they differed significantly from each other when compared via Student’s t-test. Thus, we could show that the cBSA- and BSA-fibers were smooth but differed in terms of diameter and roughness. To check the stability of the protein fibers against proteases, the fibers were incubated in a protease solution (trypsin/EDTA). The protein concentration in the supernatant of the BSA-fibers was constant after 12 min of incubation in the enzyme solution. After 60 min, a protein concentration of 152.54 ± 85.84 µg/mL (mean ± SD) was measured. In contrast, the protein concentration in the supernatant of the cBSA-fibers was 71.41 ± 11.45 µg/mL (mean ± SD) after 60 min. These experiments revealed that the cBSA-fibers showed in comparison to the BSA-fibers a higher stability against proteases like trypsin.

**Table 1:** Rq values of cBSA and BSA fibers obtained by AFM analysis.

	Measuring position	cBSA fibers, Rq [nm]	BSA fibers, Rq [nm]
Fiber A	1	0.365	0.182
	2	0.267	0.265
	3	0.314	0.185
Fiber B	1	0.23	0.166
	2	0.302	0.187
	3	0.309	0.163
Fiber C	1	0.29	0.29
	2	0.379	0.26
	3	0.235	0.187
Mean +/− SD		0.299 +/− 0.048	0.209 +/− 0.045

### Biocompatible cBSA-fibers affect MSC’s morphology

**Figure 2:**
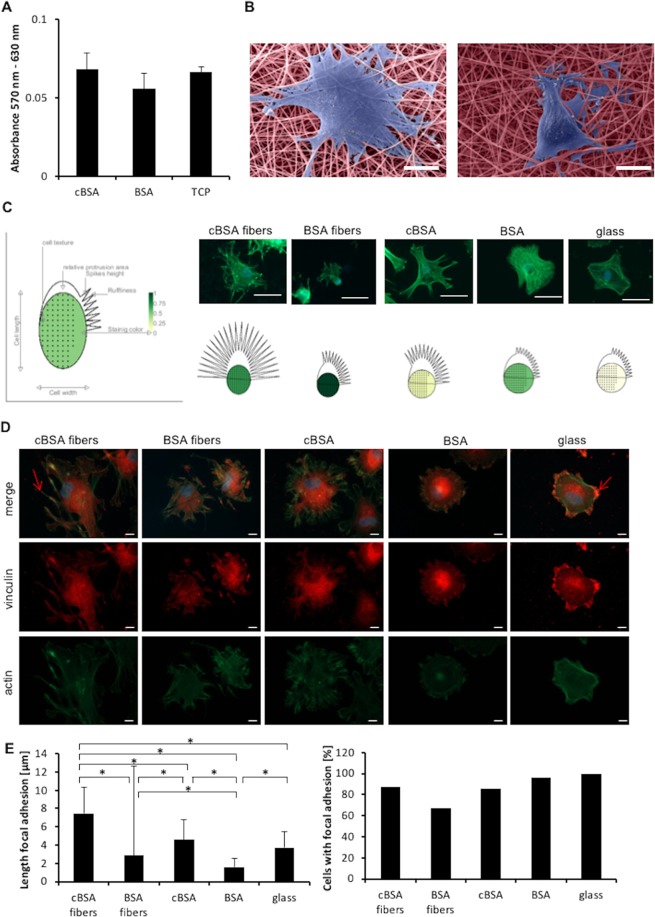
Analysis of adhesion of MSCs on cBSA- and BSA-fibers. (**A**) Bar graphs of the metabolic activity of MSCs on cBSA- and BSA-fibers and TCP. Error bars represent SE. N = 3 independent experiments. Student’s t-test was used to assess potential statistical differences. (**B**) SEM images of MSCs (violet) on cBSA- (left) and BSA-fibers (right) colored in light red. Scale bar: 20 µm. (**C**) Fluorescence microscopy images from MSCs on cBSA-fibers, BSA-fibers, cBSA-coated glass, BSA-coated glass and untreated glass. F-actin of the cytoskeleton is stained in green with phalloidin Alexa flour 488 and the nuclei are stained in blue (DAPI). Scale bar: 50 µm. Schematic drawing of cells morphology on different samples created with the Matlab plugin phenoplot (lower row). The legend of schematic drawings is shown left. N = 3 independent experiments. Bar graphs of the values included in the phenoplot are included as supplementary information in Fig. S3. (**D**) Immunofluorescence staining from MSCs on cBSA- and BSA-fibers as well as cBSA- and BSA-coated glass and untreated glass. The nuclei are stained blue (DAPI), actin filaments are stained in green and vinculin is marked in red. Top row shows an overlay picture of all channels and the lower rows show vinculin and actin staining alone. Scale bar: 10 µm. (**E**) Bar graph of quantified length of FACs in cells on different surfaces (cBSA-fibers, BSA-fibers, cBSA-coated glass, BSA-coated glass and glass) (left). Up to 10 FACs per cell were measured and the mean of the FACs from 36–60 cells with visible FACs from two different donors is plotted. Error bars represent the SD from 285–420 FACs. Statistical test: Anova with Tukey’s post-test. The percentage of cells with visible FACs (counted from 41–67 cells in total) on the different surfaces is plotted in the right bar graph. Fraction of cells displaying focal adhesions calculated from the sum of all assessed cells from N = 2 independent experiments.

For testing the biocompatibility, the fiber meshes were seeded with MSCs and the metabolic activity of cells was measured afterwards (Fig. 2A). The metabolic activity of cells cultured on cBSA- and BSA- fibers was not significantly different from the one measured for cells grown on tissue culture plastic (TCP) as assessed by Student’s t-tests. Analysis of the cells on the fibers revealed differences in cell morphology (Fig. 2B). For further investigation, F-actin in the cytoskeleton of the cells was stained. To visualize the cell morphology and the cell staining, we generated phenoplots of the cells grown on different substrates (Fig. 2C; respective bar graphs included in Fig. S3). In Fig. 2C an illustrative graph shows how the individual parameters are illustrated in the phenoplots beside. Cell length, cell texture, relative protrusion area, spikes height, ruffliness (amount of protrusions) and intensity of the staining were measured, normalized to the highest value of one parameter. The cell texture shown as dots in the graphs describes the distribution of the pixel intensities and provides information about the formation of actin bundles. Packed actin bundles can be directly linked with cellular response (e.g. cell migration)^24^. The relative protrusion area describes the area of lamellipodia, the spikes height the length of filopodia and the ruffliness is defined as amount of protrusions (the amount of drawn spikes in the graphs). Cells cultured on cBSA-fibers and cBSA-coated glass showed more and longer protrusions compared to cells cultured on other surfaces (ruffliness on cBSA-fibers 82.08 ± 11.30 and cBSA-coated glass 60.60 ± 4.72; spikes height on cBSA-fibers 12.15 ± 4.13 and cBSA-coated glass: 6.80 ± 1.52). On BSA-fibers the cells showed a cell length of 73.86 ± 11.39 μm which was smaller compared to cell length on other surfaces. Furthermore, the cells showed more and longer protrusions (ruffliness 47.90 ± 2.13; spikes height 6.51 ± 0.41) in contrast to cells on cBSA-coated glass (ruffliness 45.15 ± 4.69; spikes height 3.19 ± 0.47) or glass (ruffliness 41.75 ± 4.60; spikes height 3.23 ± 0.34). The intensity of the actin staining was higher in cells cultured on cBSA-fibers and BSA-fibers compared to cells cultured glass (green colour intensity in illustrated graphs). In line with this, the cell texture – illustrated as dots in the cell body indicating actin bundles – was higher in cells on the fibers compared to cells cultured on glass. For visualization of the focal adhesion complexes (FACs), vinculin was stained in MSCs (Fig. 2D). To investigate the impact of the different surfaces on FACs formation of single cells the length of FACs was quantified as well as the percentage of cells containing FACs (Fig. 2E). To show the distribution between the cells, the mean ± SD of the measured FACs length from 36–60 cells per condition from two different donors is plotted. MSCs on all surfaces showed diffuse staining. The vinculin bundles were longer in the cell periphery of cells cultured on cBSA-fibers (7.46 ± 2.86 μm) compared to cells cultured on all of the other surfaces (BSA-fibers: 2.89 ± 9.78 μm; cBSA: 4.57 ± 2.20 µm; BSA 1.59 ± 0.90 μm; glass: 3.74 ± 1.77 μm). On BSA-fibers 67.16% of the cells showed FACs, a value that was smaller than those measured for cells cultured on cBSA-fibers, cBSA- and BSA-coated surfaces and glass. For comparison, 85.48% of the cells cultured on cBSA revealed vinculin structures with 4.57 ± 2.20 μm in length. Cells on glass were the only sample showing small FACs (3.74 ± 1.77 μm) in all cells. In summary, both fiber types were cytocompatible and affected the cell morphology regarding FAC formation.

### cBSA-fibers support the osteogenic differentiation of MSCs

**Figure 3:**
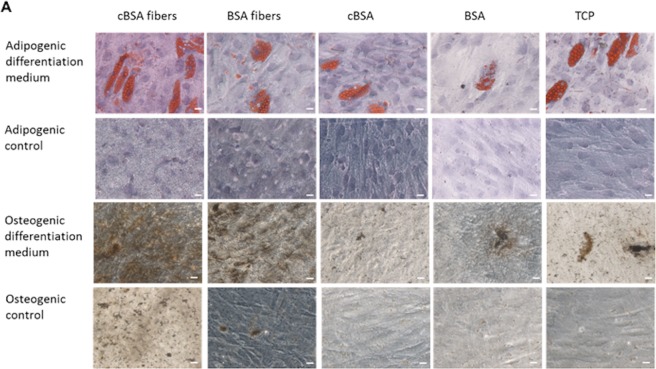
Staining of MSCs after culture on the fibers in differentiation and control media. Representative images of MSCs cultured in adipogenic differentiation medium (1st row) and control medium (2nd row) are shown. The fat vacuoles were stained with Oil red (red). In the third and fourth row, representative light micrographs of MSCs cultured in osteogenic differentiation medium (3rd row) and the appropriate control (4th row) stained with the von Kossa staining are included. The calcium-phosphate deposits are visible in brown. Scale bar: 20 µm. N = 3 independent experiments.

For investigation of adipogenic or osteogenic differentiation MSCs were cultured on cBSA- and BSA-fibers, cBSA- and BSA-coated TCP and untreated TCP in adipogenic and osteogenic cell culture medium as well as control medium. After 21 days, the cells were stained with Oil red to show adipogenic differentiation and with von Kossa staining for osteogenic differentiation (Fig. 3). In all samples cultured in adipogenic medium Oil red staining revealed fat vacuoles in the cells. The cells on the cBSA- and BSA-fibers grown in osteogenic medium showed a more pronounced staining of calcium phosphate deposits than cells in the appropriate negative control.

**Figure 4:**
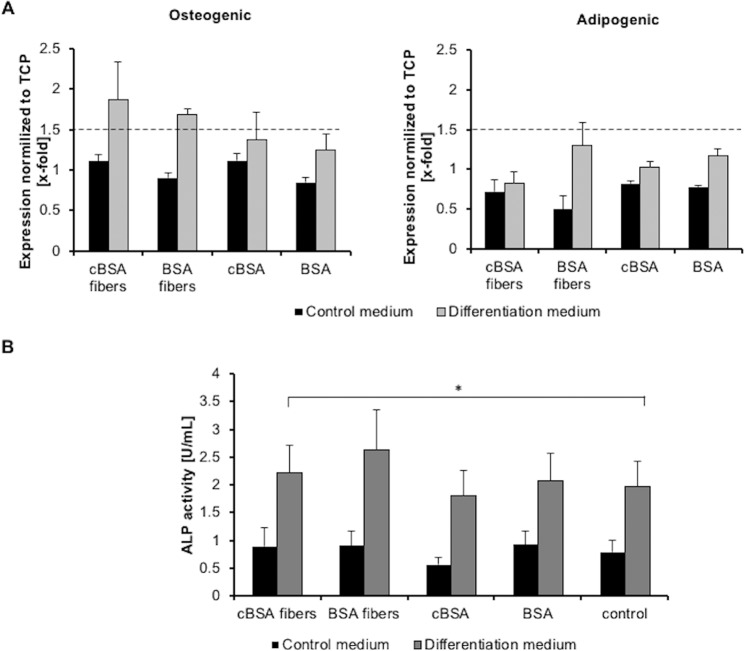
Analysis of differentiation markers in MSCs after culture on fibers. (**A**) RT-qPCR of the osteogenic (left) and adipogenic (right) differentiation marker in MSCs cultured on cBSA- and BSA-fibers as well as cBSA- and BSA-coated TCP in control medium (black) or differentiation medium (grey). The plots represent the gene expression in MSCs cultured on the different samples normalized to the expression in MSCs cultured on TCP. Dotted line represents the threshold gene expression regarded as different: 1.5-fold-change. (**B**) ALP activity in the supernatant of MSC cultures on cBSA- and BSA-fibers as well as cBSA- and BSA-coated TCP in control medium (black) or osteogenic differentiation medium (grey). The error bars represent the SE. N = 3 independent experiments. All samples were compared to the control via Student’s t-test.

Cell differentiation was further analyzed by quantification of the expression of an osteogenic (OCAL) and an adipogenic (PPARγ) marker in MSCs using RT-qPCR. The expression of the marker genes in cells cultured on cBSA-fibers, BSA-fibers, cBSA-coated TCP and BSA-coated TCP were normalized to the expression detected in cells cultured on TCP (Fig. 4A). Cells cultured on the cBSA-fibers in osteogenic differentiation medium showed 1.87 ± 0.46-fold higher OCAL expression compared to cells cultured on TCP and thereby exceeded the threshold of 1.5-fold-change indicating a verifiably higher gene expression. Cells on BSA-fibers and cBSA-coated TCP showed the same tendency and the expression of OCAL was respectively 1.68 ± 0.06-fold and 1.38 ± 0.33-fold higher than in cells grown on TCP. Cells cultured in control medium showed no increase in OCAL expression. After culture in adipogenic medium no difference in PPARγ expression in MSCs was revealed. To verify the increased osteogenic marker expression of the MSCs on cBSA-fibers, the ALP activity in the supernatant after cultivation of the cells was analyzed (Fig. 4B). The MSCs cultured on cBSA-fibers in differentiation medium showed a higher ALP activity (2.22 ± 0.48 U/mL) in the supernatant compared to 1.90 ± 0.44 U/mL ALP activity in the supernatant of cells cultured on TCP. In the supernatant of cells cultured on BSA-fibers, cBSA-coated and BSA-coated flat surfaces showed no significant increase in the ALP activity compared to the control cells cultured on TCP. Thus, only the cBSA-fibers and none of the other materials or surfaces led to statistically significant enhanced ALP activity in comparison to TCP.

### cBSA-fibers facilitate the deposition of hydroxyapatite but mineralized fibers do not support MSC differentiation

**Figure 5:**
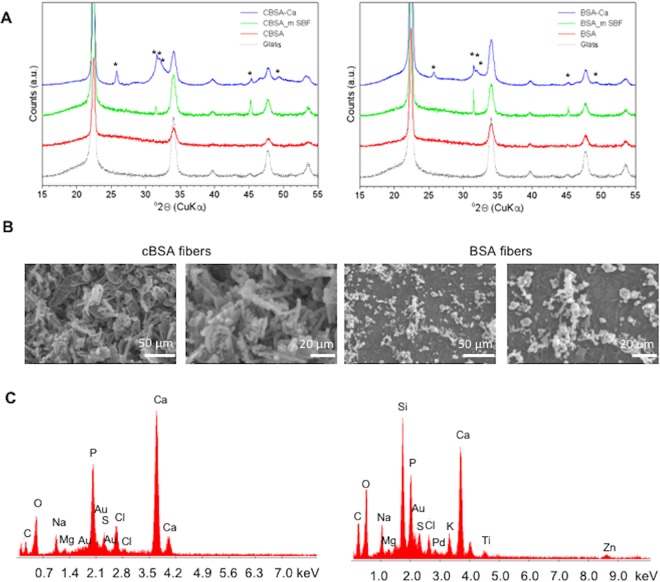
SEM imaging and EDX measurements of mineralized cBSA- and BSA-fibers. (**A**) XRD-pattern of precipitates on the cBSA- (left) and BSA-fibers (right). In these graphs the angle 2Θ is plotted against peak intensity, giving crystallographic information. The asterisks mark the characteristic peaks for hydroxyapatite. As controls, the spectra of non-mineralized cBSA-, BSA-fibers and glass are plotted. The measurement were done for one representative experiment. (**B**) SEM images of mineralized cBSA- (left) and BSA-fibers (right) using a Ca^2+^/HPO_4_^2−^ solution taken for one representative experiment are shown here. Scale bars from left to right: outer left 50 µm; inner left 20 µm, inner right 50 µm; outer right 20 µm. (**C**) EDX-spectrum of the mineralized cBSA- (left) and BSA-fibers (right) taken from one representative experiment.

During the osteogenic differentiation experiments we observed enhanced mineralization on the fibrous substrates and thus hypothesized that the calcifiability of the produced fibers might play a role for their effects on MSC differentiation. To test this hypothesis, the calcifiability of the different fibers was tested by *in vitro* mineralization. For this purpose, two protocols were tested. On one hand, the fibers were mineralized with m-SBF, a solution which mimics the ionic components of the human blood plasma. On the other hand, a highly concentrated Ca^2+^/HPO_4_^2−^ solution was used followed by an incubation in m-SBF solution. After 7, 14 and 21 days the calcium-phosphate deposits on the fibers and on glass were stained with a von Kossa staining to determine the mineralization method and time frame to achieve strongest mineralization (Fig. S4). On the fibers incubated with the Ca^2+^/HPO_4_^2−^ solution, a larger area was occupied by brown calcium-phosphate deposits compared to glass or fibers incubated solely in m-SBF. The fibers in the control solution (PBS) showed no deposits. The amount of deposits was the same after 7, 14 and 21 days of incubation. After 21 days of incubation XRD analyses were carried out (Fig. 5A). The diffraction pattern of the cBSA-fibers and BSA-fibers after incubation in a Ca^2+^/HPO_4_^2−^ solution (2θ = 25.8°, 2θ = 31.6°, 2θ = 32.1°, 2θ = 43.7°, 2θ = 46.6° und 2θ = 49.4°) showed the characteristic pattern of hydroxyapatite (2θ = 25.9°, 2θ = 31.7°, 2θ = 32.1°, 2θ = 32.8°, 2θ = 46.6° and 2θ = 49.4°)^25^. For further experiments, the fibers were incubated in Ca^2+^/HPO_4_^2−^ solution for 21 days. Out of these experiments, SEM images were taken from one representative experiment. SEM images revealed round stacked particles on the surface of the cBSA- and BSA-fibers after mineralization (Fig. 5B). These particles covered the complete surface of the cBSA-fibers, whereas the BSA-fibers were covered partially. EDX measurements (Fig. 5C) of the crystal content on the mineralized cBSA- and BSA-fibers revealed calcium (Ca) and phosphate (P). Peaks for carbon (C), oxygen (O), sodium (Na), magnesium (Mg) and chloride (Cl) were allocated to m-SBF solution and peaks for gold (Au), silicium (Si), Titanium (Ti) and Zinc (Zn) were characteristic for glass. Thus, the cBSA-fibers support the hydroxyapatite growth after using a highly concentrated Ca^2+^/HPO_4_^2−^ solution.

**Figure 6:**
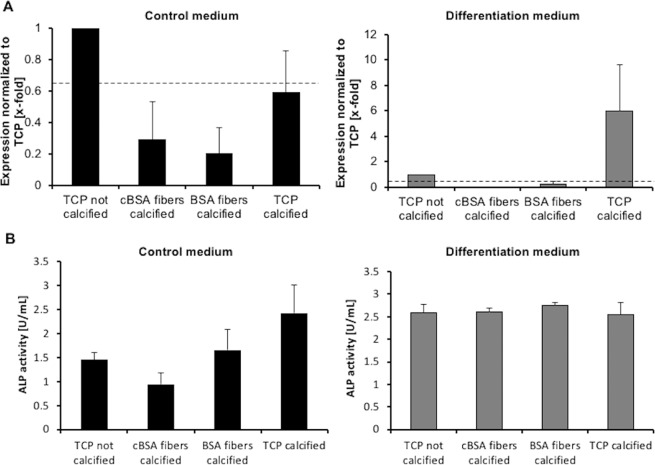
Analysis of osteogenic differentiation of MSCs cultured on mineralized surfaces. (**A**) Normalized expression of the osteogenic marker OCAL in MSCs cultured on mineralized cBSA-fibers, BSA-fibers and TCP in control medium (black, left) or differentiation medium (grey, right). Dotted line represents the threshold at 0.66-fold expression. (**B**) Plots of the ALP activity in the supernatant of cells cultures on mineralized cBSA-fiber, BSA-fibers and TCP in control medium (black, left) or in differentiation medium (grey, right) normalized to non-calcified TCP. The error bars represent the SE. N = 3 independent experiments. All samples were compared to the control via Student’s t-test.

To investigate the impact of the mineralization of the fibers on the osteogenic differentiation of MSCs, the cells were cultured on *in vitro* mineralized cBSA-fibers, BSA-fibers, and untreated TCP (Fig. 6A+B). The expression of the osteogenic marker OCAL in MSCs grown on mineralized cBSA-fibers, BSA-fibers and calcified TCP in control medium were lower than on the non-mineralized TCP (Fig. 6A). In osteogenic differentiation medium only the MSCs cultured on calcified TCP showed a 6.00 ± 3.62-fold higher OCAL expression than cells cultured on non-mineralized TCP. The surface of TCP was not completely covered with hydroxyapatite crystals in comparison to fully mineralized cBSA-fibers. In the control medium, the ALP activity of the cells cultured on mineralized cBSA-fibers was lower than that of the cells cultured on non-calcified TCP. At the same time the ALP activity of cells grown on BSA-fibers and calcified TCP was slightly higher than on non-calcified TCP. However, none of these effects was statistically significant. In the differentiation medium, the ALP activity after culture on mineralized cBSA-fibers, BSA-fibers and TCP was similar to non-mineralized TCP. In summary, no enhanced osteogenic MSC differentiation on the mineralized fibers was detected.

## Discussion

Broadening the field of tissue engineering in a context of supporting bone repair and regeneration in a clinical setting, needs development of new and innovative materials. The ideal biomaterial for such applications would either incorporate MSCs, which eventually differentiate into bone cells, or would be able to enhance bone regeneration via growth factor delivery or guiding structures to influence MSCs in the material’s vicinity *in vivo*. A prerequisite to reach this goal, is a fundamental understanding of how MSC’s behavior is influenced by chemical and physical properties of the applicable materials. Taking a step forward in this direction, we developed an *in vitro* system resembling the fibrous structure of structural proteins and the adhesive nature the ECM in some properties which allows the analysis of cell-material interactions and their impact on MSC differentiation.

In the current study, fibers were generated using cBSA and BSA. Cationization of BSA was achieved by amination of the carboxyl groups of the protein (shown by MALDI TOF measurements) similar to studies from the Weil group^26^. These fibers were characterized based on their analogy to *in vivo* tissue and their suitability for cell culture and cell studies. In *in vivo* conditions, the bone marrow and the bone matrix is traversed by fibrous ECM proteins such as collagen^27,28^. The cost-effective cBSA- and BSA-fibers showed a diameter of 1.53 µm and 2.15 µm respectively which were similar in size to natural collagen fibers^29,30^. The observed difference in fiber diameter appears to be dependent on the viscosity of the polymer solution as shown in a previous study which determined that the viscosity of a polymer solution had an impact on the diameter of electrospun fibers^31^. Both fiber types had smooth surfaces with Rq values in the nanometer range. However, despite being low, the obtained values for the two fiber types differed slightly from each other. Surface roughness is known to influence the osteogenic differentiation of MSCs at the micrometer-scale^32–34^. Thus we assume that the small differences in surface roughness of the developed fiber types are too small to affect osteogenic differentiation of MSCs.

Furthermore, the cBSA-fibers with the lower diameter were more resistant against proteases in cell culture in comparison to BSA-fibers. Both fibers were cytocompatible and therefore suitable as cell culture template. In comparison to a recent study in which a globular albumin-polyethyleneoxide mixture was used, we showed different results, namely that the cBSA-fibers as well as BSA-fibers were cell-adhesive^35^. Based on the results of a previous study which showed that positively charged amine groups facilitated cell adhesion, we expected that the prior to denaturation added amine groups were exposed, favoring cell adhesion^36^. With these properties, the developed fibers have advantages over other frequently used proteins such as collagen, which shows certain limitations as backbone for scaffolds in cell cultures: (i) high costs of manufacturing, (ii) degradation of the costly protein into gelatin during electrospinning which can affect adhesion sites for cells, (iii) low mechanical strength and resistance against proteases such as naturally expressed collagenases which can destroy the scaffold structure and (iv) low osteoinductivity^22,37–39^. The developed *in vitro* system in comparison is cheap in manufacturing, resembles the fibrous structure of protein fibers in the ECM, allows adhesion and cultivation of MSCs due to its resistance against proteinases.

To analyze the impact of the fibers on MSCs’ adhesive behavior and their differentiation regarding osteogenic induction, MSCs were seeded on cBSA- and BSA-fibers as well as cBSA- and BSA-treated surfaces. The MSCs on cBSA-fibers showed stress fibers characterized by pronounced actin bundles and long cellular protrusions with FACs. On these cationized fibers, MSCs displayed the longest FACs in comparison to all other tested surfaces, including cBSA-coated surfaces without fibers and BSA-fibers which were not aminated and thus cationized. This result indicates that the charge of the fibers and the fibrous topography might influence MSC adhesion. This hypothesis is supported by the findings (i.) that MSCs on non-fibrous cBSA-coated surfaces had longer FACs than on BSA-coated surfaces, which shows that the cationization of the BSA influences the analyzed cell function and (ii.) that FACs found in MSCs on BSA-fibers were longer than on pure BSA which substantiates that the fibrous structure enhances FAC formation by MSCs. This is similar to previous results with electrostatic spun collagen fibers in which the MSCs formed adhesion contacts depending on the fibrous topography of the surface^40^. In line with our obtained results, Rebl and colleagues also reported an increased vinculin contact length on amino group-covered (cationized) synthetic polymer fibers^41^. Our results indicate that the positively charged surface and the fibrous topography elicited by the shape of the fibers effect MSC adhesion and their cytoskeleton rearrangement. Previously published results which showed that increased cell spreading facilitated the osteogenic differentiation of MSCs led us to conclude that our material could also favor MSC differentiation^42^.

The differentiation process of MSCs into bone forming osteoblasts is known to be controlled by various parameters. The surface topography and charge of a cell culture substrate can not only enhance the focal adhesion but also increase osteogenic MSC differentiation^42–46^. In line with these studies, we could show a tendency of higher expression of the osteogenic marker OCAL in MSCs as well as a higher ALP activity in the supernatant after culture on cBSA-fibers concluding an increased osteogenic differentiation. The adipogenic differentiation seemed not to be effected. Furthermore, a large amount of hydroxyapatite crystals was visible on the charged fibers after MSC culture. The hypothesis that the large number of amino groups of the cBSA-fibers act as nucleation points for mineralization was verified by *in vitro* mineralization of the fibers which revealed a higher amount of hydroxyapatite crystals on cBSA-fibers in comparison to BSA-fibers. By working in a sterile, largely dust-free environment and the comparison of BSA- and cBSA-fibers, which have similarly high surface areas, these factors could be excluded as possible reasons for the observed enhanced mineralization on cBSA-fibers. In conjunction with a previous study showing that charged materials can act as nucleation points for mineralization^47^, we conclude that the charged amino groups introduced by cationization into the cBSA can serve as nucleation points to favor mineralization. Interestingly, despite several studies showing that *in vitro* mineralized scaffolds including fibrous scaffolds support the osteogenic differentiation of MSCs, we obtained contradictory results^10,48,49^. Our experiments revealed that *in vitro* mineralization of the cBSA-fibers which covered the entire growth surface did not support the osteogenic differentiation to a higher extent than TCP characterized by an increase of the osteogenic marker OCAL or the ALP activity. This finding suggests that the modification of the fibers facilitated the deposition of hydroxyapatite similar to collagen *in vivo*^6^. The cationization of the fibers as well as the fibrous topography of the cell culture substrates, supported the osteogenic differentiation in contrast to fully mineralized fibers in which the charged nucleation points and fibrous structure were completely covered. A recent study found that increased surface charge promotes attachment of proteins resulting in increased MSC adhesion and potentially affected MSC differentiation^50^. More specifically, cationized materials had been shown to be beneficial for the adsorption of expressed proteins^51^. This supports the assumption that differentiation-supporting soluble factors such as osteogenic factors in the cell culture medium could adsorb to the cationic fibers which can be a benefit for MSC differentiation and that this effect was minimized when the fibers were covered with hydroxyapatite. In summary, the differentiation process of MSCs on the fibers was controlled by the fibrous topography as well as the cationization of the fibers and it seemed that this process was associated with a rearrangement of the cytoskeleton and formation of FACs.

In conclusion, we developed a new scaffold of pure protein and investigated the effect of its fibrous topography and charge on the differentiation of MSCs. Our results revealed the effect of fibrous structure and surface charge on osteogenic differentiation of MSCs and deposition of bone composite hydroxyapatite. The developed protein fiber system is a versatile tool resembling the fibrous structure of protein fibers in the ECM and allowing chemical and topographical modification, which can be used as model system for fundamental studies to understand how MSCs are guided into osteogenic differentiation for *in vitro* investigation. Unfolding properties such as the charge or topography, which can positively affect MSC behavior, can help to improve surface modifications of osteoinductive implants.

## Material and Methods

### Synthesis of cationized bovine serum albumin (cBSA) and fiber production

To improve the cell adhesive properties to bovine serum albumin (BSA) we introduced amino groups into the protein. For this purpose, 600 mg BSA was dissolved in a 50 mM ethylenediamine solution (Sigma-Aldrich, St. Louis, Missouri, USA. pH 4.75) with 3.067 g *N*-(3-dimethylaminopropyl)-*N*′-ethylcarbodiimide hydrochlorid (EDC, Sigma-Aldrich). The solution was stirred at 160 rpm for 2 h at room temperature. The reaction was stopped by adding 4 mL 4 M acetate buffer (2 M acetic acid, 2 M sodium-acetat (Sigma-Aldrich), pH 4,75). The cationized BSA (cBSA) was washed two times with 100 mM acetate buffer and 5 times with ddH_2_O in a 20 kDa Vivaspin® concentrator (Sartorius, Göttingen, Germany). Afterwards, the cBSA was freeze dried (Alpha 1–4 LD plus Freeze dryer, Martin Christ, Osterode am Harz, Germany) and stored at −20 °C until further use. The success of cationization of the protein was analyzed MALDI-TOF mass spectrometry (Bruker Autoflex III, Billerica, Massachusetts, USA). For this purpose, 1 mg/mL BSA or cBSA were dissolved in acetonitrile/water (1:1) and mixed 1:5 with the matrix (10 mg/mL sinapinic acid in acetonitrile/water). 1 µL of this solution was spotted onto the target plate and measured after drying of the sample.

The fibers were generated by using the electrospinning technique. For this purpose, 200 mg cBSA or 100 mg BSA were diluted in 900 µL 2.2.2-trifluorethanol (Sigma-Aldrich), 84.1 µL ddH_2_O and 17.9 µL β-mercaptoethanol (Sigma-Aldrich). The protocol is based on the publication from Dror *et al*.^52^. The solution was transferred to a 1 mL syringe (Inject®F., B.Braun, Melsungen, Germany) with a blunt needle as spinneret (Stainlees Steel Blunt Needle, Component Supply GmbH, Wolfsburg, Germany). The syringe was installed in a syringe driver (Landgraf Laborsysteme GmbH HLL, Langenhagen, Germany) with 12 cm distance from the collector and the fibers were generated with a flow rate of 0.5 mL/h and 16 kV. On the collector round glass slides (15 mm, Carl Roth, Karlsruhe, Germany) were placed collecting the fibers.

### Measurement of the fiber diameter

Images of the cBSA- and BSA-fibers were taken with a VHX Digital microscope (Keyence, Osaka, Japan). For the analysis with the software VHX 5000 (Version 1.5.1.0) 10 images of each fiber type from 3 different batches were taken. We measured the diameter of 5 different fibers in one image. In total 150 data points were generated for cBSA and BSA each.

### Atomic force microscope (AFM) measurements

AFM imaging for roughness measurements was done on a Dimension Icon setup (Bruker, Germany) in tapping mode with HQ:NSC15/Al BS cantilevers (MikroMasch, Germany), having a nominal force constant of 40 N/m and a resonance frequency of 325 kHz. To exclude the influence of the curvature, image size was kept at 50 nm × 50 nm (see Supplementary information, Fig. S2) and for each fiber type, nine different positions on three different fibers were imaged (resolution of 512 lines, 2 Hz scan frequency). The images were then analyzed for Rq with the onboard software without further treatment.

### Sterilization of the fibers

The fibers on 15 mm glass slides were placed in a 24-well cell culture plate (Greiner BioOne, Frickenhausen, Germany) and washed 3 times with 1 mL phosphate buffered saline (PBS, Sigma-Aldrich) for 10 min each. Afterwards, the fibers were sterilized for 10 min with 70% (v/v) ethanol. To get rid of ethanol residues the fibers were washed 3 times with 1 mL PBS.

### Degradation of the fibers

The sterilized cBSA- and BSA-fibers were placed in a 24-well cell culture plate and 1 mL trypsin/EDTA solution (Sigma-Aldrich) was added to the wells. Additionally, 1 mL trypsin/EDTA solution was added to an empty well as control. The samples were incubated for 3 min, 6 min, 12 min, 20 min and 60 min at 37 °C and 5% CO_2_. At every time point the protein concentration in 25 µL of the supernatant was measured using the Pierce™ BCA Protein Kit (Thermo Fisher Scientific, Waltham, Massachusetts, USA) at 562 nm (EnSpire Multimode Plate Reader PerkinElmer, Rodgau, Germany). The protein concentration of the control was subtracted from the one of the samples.

### Calcification of the fibers

The applied mineralization protocol of the fibers is based on the publications of Shih *et al*. and Phadke *et al*.^10,47,53^. The cBSA- and BSA-fibers or the glass slides (control) were placed in a 12-well cell culture plate and washed three times with PBS for 2 h each. Afterwards, 1 mL modified simulated body fluid (m-SBF, containing 142 mM Na+, 5 mM K^+^, 1.5 mM Mg^2+^, 2.5 mMCa^2+^, 103 mM Cl^−^, 10 mM HCO_3_^−^, 1 mM HPO_4_^2−^ and 0.5 mM SO_4_^2−^) was added to the slides and incubated for 12 h. m-SBF mimics the human blood plasma and it has been shown that the ion concentration of this solution was optimal for *in vitro* production of bone-like hydroxyapatite. We followed the protocol of Oyane *et al*. for m-SBF preparation^54^. To improve the mineralization, the fibers were incubated afterwards in 1 mL of a 0.04 M Ca^2+^ (Merck Millipore, Darmstadt, Germany)/0.024 PO_4_^3−^ (Merck Millipore) solution (pH 5.2) for 1 h at 37 °C. To determine the calcification time, kinetics of fiber mineralization was recorded in one initial experiment. For this purpose, the samples were incubated m-SBF solution at 37 °C. The solution was changed every 2 to 3 days and von Kossa staining of the samples was performed after 7, 14 and 21 days of incubation. For subsequent cell culture experiments the samples were incubated in m-SBF for 21 days after treatment with the 0.04 M Ca^2+^ 0.024 PO_4_^3−^ solution. Before the cell culture experiments the mineralization of the fibers after 21 days of incubation was visually assessed via light microscopy (Axiovert A1 light microscope, Carl Zeiss, Oberkochen, Germany) and only fibers that showed mineralization similar to the results in Fig. S2 were taken for the subsequent experiments.

### Scanning electron microscopy (SEM) of fibers with and without cells

For scanning electron microscopy (SEM), the cBSA- and BSA-fibers colonized with MSCs were fixed with 2.5% glutaraldehyde for 10 min and dehydrated in an increasing ethanol series (50% (v/v), 60% (v/v), 70% (v/v), 80% (v/v), 90% (v/v), two times 100% (v/v)) for 10 min each. Afterwards, the samples were frozen at −80 °C in 100% (v/v) ethanol and freeze-dried for 24 h (Alpha1–4, Martin Christ Gefriertrocknungsanlagen, Osterode am Harz, Germany). Scanning electron micrographs were obtained, using a Philips XL 30 FEG ESEM operated at an acceleration voltage of 10 kV at high vacuum or 20 kV at a chamber pressure of 133 Pa (1 Torr). To improve image quality, the fibers with and without cells were sputtered with a thin conductive layer (5 nm Au/Pd 80/20) using a BAL-TEC MED020 coating system. SEM images were pseudo-colored by using PS Adobe Photoshop CS5 Extended Version 12 (Dublin, Ireland). For energy dispersive x-ray (EDX) elemental analysis of the mineralized fibers a liquid nitrogen cooled Sapphire Si(Li) detecting unit from EDAX (Mahwah, USA) was used. For spectra collection, the microscope was operated at 20 kV using a 100 µm aperture. The measurement time was 120 s.

### X-ray diffraction (XRD) measurements

A Bruker D8 Advance A25 diffractometer (Bruker AXS GmbH, Karlsruhe, Germany) equipped with a LYNXEYE XE Detector (opening degree 2.94° and 192 channels) was used for XRD analysis of the fibers. Patterns were recorded between 3 and 60° 2θ (CuKα radiation), with a counting time of 3.5 s and a step size of 0.02° 2θ, a fixed slit of 0.18°, soller collimator of 2.5° (primary and secondary side) and a knife edge 3 mm above the specimen holder.

### Cell culture

Bone marrow-derived MSCs were provided by Prof. Dr. Karen Bieback and cultured in DMEM supplemented with 5% platelet lysate (PL) and 2 U/mL heparin (PL Bioscience, Aachen, Germany)^55^. The cells were subcultured at 80% confluency, seeded in a density of 2000 cells/cm^2^ and cultured under standard conditions at 37 °C, 5% CO_2_. Only MSCs from passage 2 to 6 were subjected to experiments.

### Immunofluorescence staining of MSCs

After sterilization of the cBSA- and BSA-fibers the glass slides were placed in a 12-well cell culture plate. Additionally, one well each was treated with 1 mg/mL cBSA in PBS and 1 mg/mL BSA in PBS and incubated for 1 h at 37 °C. Afterwards, the solution was removed and 2 mL DMEM with 1% (v/v) PL and 2 U/mL heparin was added into each well. 1 × 10^4^ MSCs in 100 µL medium were pipetted carefully on the different materials and incubated for 3 h at 37 °C and 5% CO_2_. The medium was removed and 500 µL 4% (w/v) paraformaldehyde (Sigma-Aldrich) in PBS was added and incubated for 10 min at room temperature. Afterwards, the cells were washed with 500 µL PBS. For permeabilization of the cell membrane, the samples were incubated in 500 µL 0.1% (w/v) Triton X-100 (Applichem) in PBS for 10 min and subsequently washed three times in PBS for 2 min. Unspecific binding of the antibody was blocked by incubating the samples in 0.2 mg/mL (100 U/mL) heparin (Porcine Intestinal Mucosa, Calbiochem®, Merck Millipore) in PBS. For immunofluorescent staining the mouse anti-human Vinculin antibody (clone: hVIN-1, Sigma Aldrich) was diluted 1:400 in 0.1% (w/v) BSA in PBS together with 1:1000 DAPI and incubated for 1 h at room temperature. The secondary antibody goat anti-mouse IgG Alexa 546 (Invitrogen^TM^, Thermo Fisher Scientific) was diluted in 0.1% (w/v) BSA in PBS together with 1:40 phalloidin Alexa 488 (Invitrogen^TM^, Thermo Fisher Scientific) and incubated for 1 h at room temperature. The samples were imaged with an inverse fluorescence microscope (AxioObserver.Z1 inverse, Carl Zeiss, Oberkochen, Germany) and analyzed with the software ImageJ (Wayne Rasband, National Institute of Health, Maryland, USA). Processing of the entire sample images and their appropriate controls is applied equally with the software Zen2012 blue (Carl Zeiss). The data were normalized to the largest value and added to Plugin Phenoplot in Matlab R2017a (Mathworks, Natick, Massachusetts, USA). The Phenoplot analysis was based on the publication of Sailem *et al*.^56^.

### Differentiation assay of MSCs on the fibers

For the adipogenic differentiation 1 × 10^5^ MSCs were seeded on two each of cBSA-fibers, BSA-fibers, 1 mg/mL cBSA-coated and 1 mg/mL BSA-coated wells of a 24-well cell culture plate as well as on untreated cell culture plate. All samples were incubated in DMEM supplemented with 5% (v/v) PL and 2 U/mL heparin for 24 h at 37 °C 5°% CO_2_. Afterwards, the medium of one of each sample was replaced by adipogenic induction medium (hMSC Adipogenic BulletKit, Lonza, Basel, Swizerland). The cells were incubated in the induction medium for 3 days followed by 2–3 days of incubation in maintenance medium. Three cycles of induction/maintenance stimulated the adipogenic differentiation of the cells. The medium of the other samples (control samples) was replaced by adipogenic maintenance medium (hMSC Adipogenic BulletKit, Lonza) and incubated for 3 days. Afterwards, the medium was changed with fresh maintenance medium and incubated for 2–3 days. This cycle was repeated three times.

For the osteogenic differentiation, 1 × 10^4^ MSCs were seeded as described above. The cells were cultured in DMEM supplemented with 5% (v/v) PL and 2 U/mL heparin at 37 °C, 5°% CO_2_. After 24 h the medium of one sample each was replaced with osteogenic medium (hMSC Osteogenic BulletKit, Lonza) and the other samples with DMEM supplemented with 5% (v/v) PL and 2 U/mL heparin. The medium was changed every 2–3 days.

### Von Kossa staining

For visualization of the osteogenic differentiation the calcium-phosphate deposits were stained by the von Kossa staining. For this the samples were fixed with 10% (v/v) formaldehyde (Applichem, Darmstadt, Germany) for 15 min. Afterwards the cells were washed with ddH_2_O and incubated in 5% (w/v) AgNO_3_ (Merck, Darmstadt) in ddH_2_O for 15 min. The cells were washed again and incubated with 1% (w/v) pyrogallol (Merck) in ddH_2_O for 2 min in a dark chamber. After a washing step, the cells were fixed with 5% (w/v) sodium thiosulfate (Sigma-Aldrich) in ddH_2_O. The samples were visualized with an Axiovert A1 light microscope adapted with a camera (Axiocam Cc1 60N-C1″1.0x camera, Carl Zeiss).

### Oil red staining

The fat vacuoles in adipogenic differentiated MSCs were stained with an Oil red staining. For this purpose, the cells were fixed in 10% (v/v) formalaldehyde for 30 min. After washing, the samples with ddH_2_O, the cells were incubated for 2 min in 60% (v/v) isopropanol (VWR, Darmstadt, Germany). Afterwards, the solution was replaced by an Oil red (Sigma-Aldrich) solution and incubated for 5 min. The Oil red solution was made up as followed: 6 ml of a 0.3% (w/v) oil red in isopropanol stock solution was diluted in 4 mL ddH_2_O. After washing, the cells Harris modified hematoxylin (Sigma-Aldrich) was added and incubated for 2 min. The fat vacuoles appeared red and the cytoplasma and nucleus violet. The cells were imaged in ddH_2_O with an Axiovert A1 light microscope adapted with a Axiocam Cc1 60N-C1″1.0x camera.

### Alkaline phosphatase (ALP) assay

For determination of the ALP activity in the cell culture medium, the ALP assay kit (Abcam, Cambridge, United Kingdom) was used. The samples were centrifuged at 300 x g for 5 min and the supernatant was used for the ALP assay. The assay procedure was done according to manufacturer’s instructions.

### RNA isolation

The cells were harvested from the different conditions and centrifuged for 5 min at 300 x g. Afterwards the cell pellet was resuspended in PBS and again centrifuged for 5 min at 300 x g. The supernatant was discarded and the cell pellet was used in the RNeasy micro isolations kit (Qiagen, Hilden, Germany) according to manufacturer’s instructions.

### cDNA synthesis and RT-qPCR

The RNA isolated from the MSCs was converted into cDNA using a reverse transcriptase (TaqMan Reverse Transcription Kit, Life Technologies). The expression of the osteogenic marker osteocalcin and the adipogenic marker PPARγ were checked by qPCR (Table 2). For this purpose, the cDNA was applied in a RT-qPCR using Kapa SYBR® Fast kit (PeqLab, Erlangen, Germany). The RT-qPCR was performed in the CFX Connect™ Real-Time PCR Detection System (BioRad Laboratories, California, US) and the following protocol was carried out: initial denaturation and enzyme activation at 95 °C for 3 min followed by 40 cycles of 95 °C for 15 sec, 58 °C for 60 sec and 65 °C for 5 sec and 95 °C for 30 sec. The Ct values were determined with the help of CFX Manager^TM^ software 3.1 (BioRad) and normalized to the housekeeping genes B2M and YWHAZ. Gene expression was calculated by using the 2^−ΔΔCT^ method.

**Table 2:** Primer sequences for the target transcripts.

Gene	Sequence (5′ → 3′)	Product length [bp]
OCAL	GAAGAGACCCAGGCGCTAC	88
	CTCACACACCTCCCTCCTG	
PPARγ	ACTTTGGGATCAGCTCCGTG	208
	GCAGGCTCCACTTTGATTGC	
B2M	TGCTGTCTCCATGTTTGATGTATCT	86
	TCTCTGCTCCCCACCTCTAAGT	
YWHAZ	ACTTTTGGTACATTGTGGCTTCAA	94
	CCGCCAGGACAAACCAGTAT	

### 3-(4,5-dimethylthiazol-2-yl)-2,5-diphenyltetrazolium bromide (MTT) assay

To check the metabolic activity of MSCs on the fibers, 50 000 MSCs were seeded on the cBSA- and BSA-fibers with 1 mL MSC growth medium and incubated for 24 h under standard cell culture conditions (37 °C, 5% CO_2_, humidified atmosphere). Thereafter, 100 µL of 5 mg/mL MTT reagent (Sigma Aldrich) in PBS were added, resuspended and incubated for 4 h under standard cell culture conditions. For cell lysis, the medium was removed and replaced by 1 mL lysis-solution (0.6% (v/v) acetic acid (Sigma-Aldrich) and 10% (w/v) SDS (Sigma-Aldrich) in DMSO (AppliChem)). The lysis solution was incubated for 30 min. Thereafter, the absorption at 570 nm and 630 nm (EnSpire Multimode Plate Reader) was measured. For this purpose, 100 µL of the supernatant (in duplicate) was used.

### Data analysis

For statistical analysis and graphical representation of the data, the software MS Excel (Microsoft, Redmond, Washington, USA) and GraphPad Prism (Graphpad Software, Inc., La Jolla, USA) were used. In repeated experiments with cells from N = 3 donors, the mean ± standard error (SE) was determined. SE (of the mean) was used to estimate the dispersion of donor means around the mean of all donors. A two tailed paired Student’s t-test was performed to assess potential significant differences between the sample and control. In differentiation experiments, which were conducted to test the potential of the developed materials to enhance MSC differentiation in comparison to the gold standard cell culture material, which is tissue culture plastic (TCP), TCP was always included as a control and the results obtained on the different materials and surfaces were compared to this control and not all of them with each other. The standard deviation (SD) was calculated in experiments, in which donor-to-donor-variation did not play a role, to show the distribution of single values. To determine significant differences between several conditions one-way Anova with Tukey’s multiple comparisons test was done with N > 3 measured values. We indicated the used statistical test in the figure legend. P-values ≤ 0.05 were regarded as statistically significant and marked with an asterisk “*”. Data without significant differences were not specially marked.

## Supplementary information

Supplementary Information

## Data Availability

The datasets generated during and/or analysed during the current study are available from the corresponding author on reasonable request.
