# Supplementary material for: Potential of electrospun cationic BSA fibers to guide osteogenic MSC differentiation via surface charge and fibrous topography
Source: Sci Rep. 2019 Dec 27;9:20003. doi: 10.1038/s41598-019-56508-6 (PMC6934613; doi:10.1038/s41598-019-56508-6)
Supplement: Supplementary file 1 — Supplementary Information [file 41598_2019_56508_MOESM1_ESM.pdf]

Supplementary Information

Potential of electrospun cationic BSA fibers to guide osteogenic MSC differentiation via surface charge and fibrous topography

Annamarija Raic, Frank Friedrich, Domenic Kratzer, Karen Bieback, Joerg Lahann, Cornelia Lee-Thedieck*

Affiliations
Dr. A. Raic, Dr. D. Kratzer, Prof. Dr. J. Lahann. Karlsruhe Institute of Technology (KIT), Institute of Functional Interfaces, Eggenstein-Leopoldshafen, 76344, Germany.

Dr. F. Friedrich. Karlsruhe Institute of Technology (KIT), Competence Center for Material Moisture, Eggenstein-Leopoldshafen, 76344, Germany.

Dr. A. Raic, Dr. D. Kratzer, Prof. Dr. C. Lee-Thedieck. Leibniz University Hannover, Institute of Cell Biology and Biophysics, Hannover, 30419, Germany.

Prof. Dr. K. Bieback. Institute of Transfusion Medicine and Immunology, Medical Faculty Mannheim, Heidelberg University; German Red Cross Blood Service Baden-Württemberg – Hessen, Friedrich-Ebert Str. 107, Mannheim, 68167, Germany.

Prof. Dr. J. Lahann. Biointerfaces Institute and Departments of Chemical Engineering, Materials Science and Engineering, Macromolecular Science and Engineering and Biomedical Engineering, University of Michigan, Ann Arbor, MI, 48109, USA.

Corresponding author
* Prof. Dr. Cornelia Lee-Thedieck, Leibniz University Hannover, Institute of Cell Biology and Biophysics, Herrenhäuser Str. 2, 30419 Hannover. Phone: +49 511 762 5629; Fax: +49 511 762 2606; Email: lee-thedieck@cell.uni-hannover.de

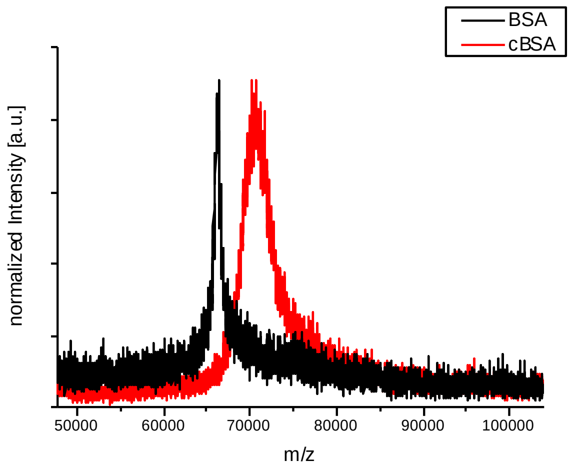

Figure S1. MALDI-TOF measurement of BSA and cBSA. The molar mass of BSA (black) and cBSA (red) was measured by MALDI-TOF mass spectrometry. In the graph the intensity is plotted versus the ratio of mass to charge (m/z).

AFM measurements of the surface roughness Rq:

In order to determine the surface roughness, three different fibers of each fiber type were analyzed at three different positions each (Table X). The scan areas were set to 50 nm × 50 nm for all measurements to make sure that only flat areas were investigated. The chosen scan area was small enough to neglect the natural transverse curvature of the individual fibers but large enough to obtain reliable information on the surface roughness over relevant surface areas. With a larger scan area, the height difference due to curvature of the fiber would have a tremendous impact on the calculation of the surface roughness and, therefore, the corresponding Rq values would deviate towards unrealistic high numbers. By choosing a scan size of 50 nm × 50 nm, the surface roughness values could be directly extracted from the height profiles without further manipulation of the obtained images such as mathematical flattening. Figure S2 displays representative height profiles obtained by AFM analysis of cBSA fibers (Figure S2A) and BSA fibers (Figure S2B).

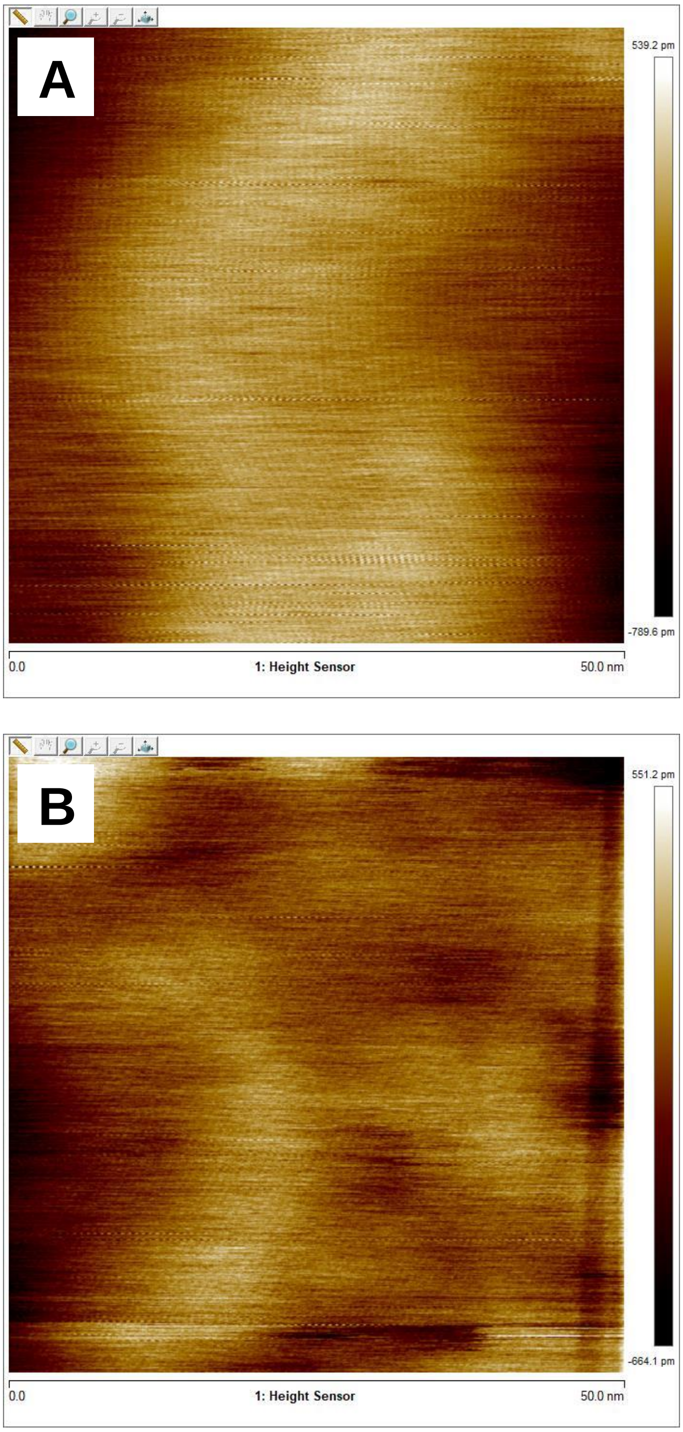

Figure S2. Representative AFM images. (A) Height profile of a cBSA fiber within a scan area of 50nm x 50 nm. (B) Corresponding height profile a BSA fiber. The Rq values given in table 2 were extracted from this series of experiments.

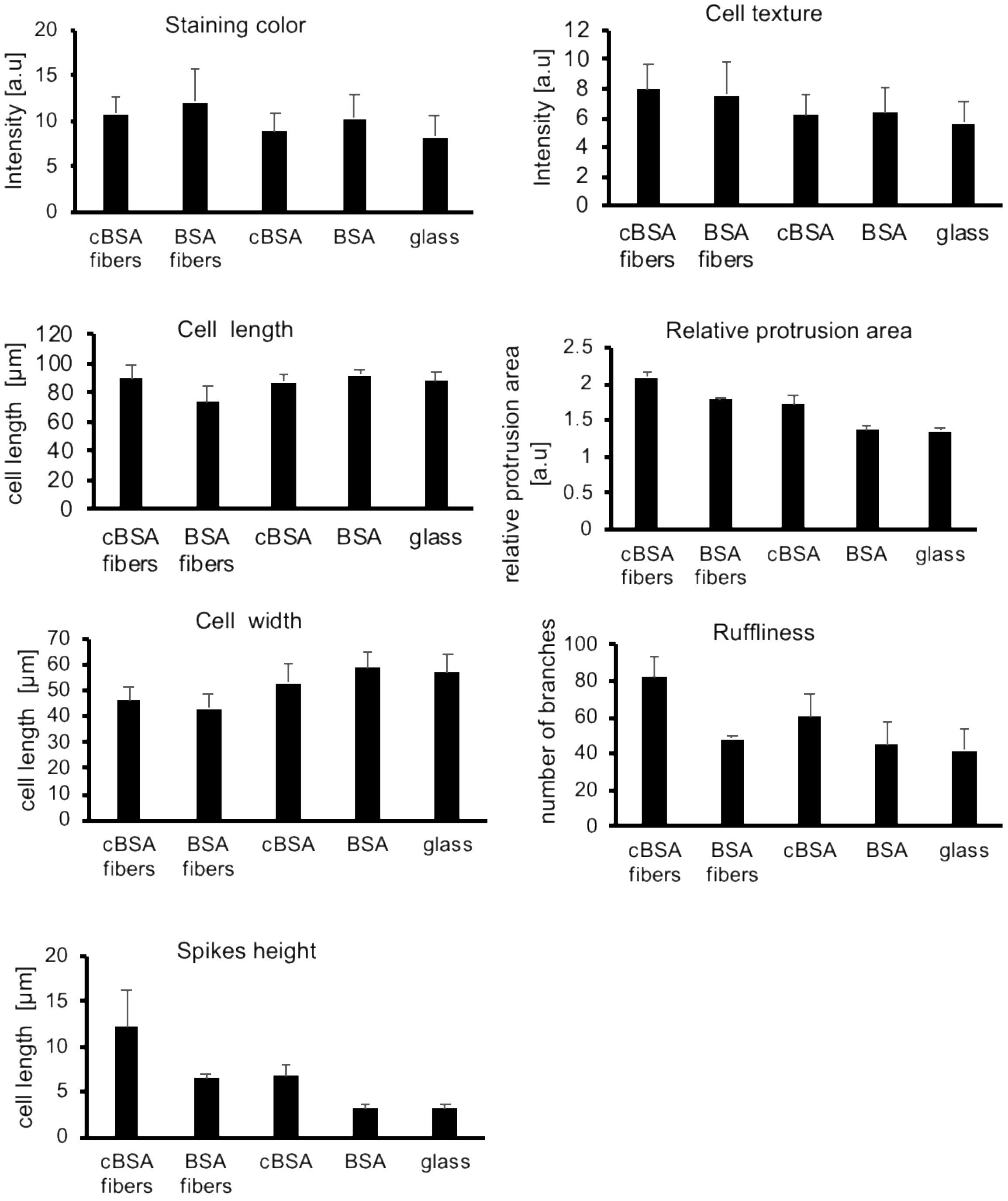

Figure S3. Quantitative analysis of cells on cBSA-fibers, BSA-fibers, cBSA-and BSA treated surfaces and glass. Cells cultured on cBSA-fibers, BSA-fibers, cBSA-and BSA treated surfaces and glass were stained with phalloidin-alexa488 and analyzed with ImageJ. The bar graphs represent the staining color, cell texture, cell length, relative protrusion area, cell width, ruffliness and spikes height of MSCs. Error bars represent the SE. Data were used to generate phenoplots of the cells.

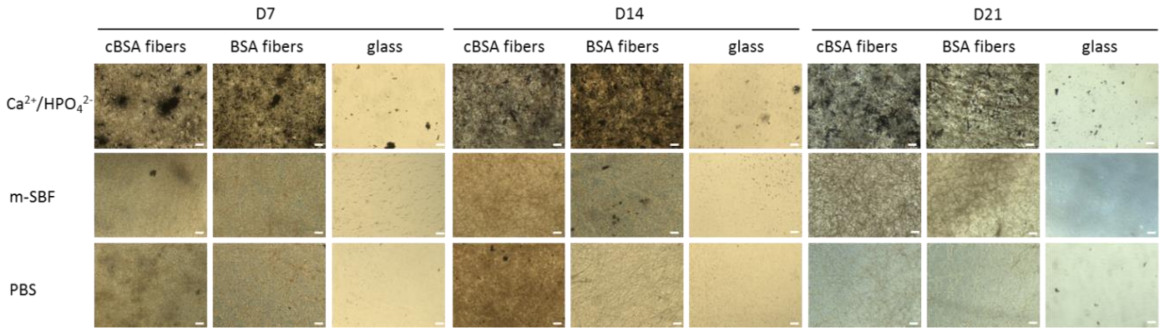

Figure S4. Mineralization of the cBSA- and BSA-fibers. (A) cBSA-, BSA-fibers and glass were treated with a Ca^{2+}/HPO_4^{2-} solution and m-SBF (first row). In addition samples were incubated in m-SBF (second row) or PBS (third row) for 7, 14 and 21 days (D7, D14, D21).The time series was carried out in one initial experiment to determine the appropriate incubation time for mineralization that was then used in subsequent experiments. The samples were stained with Van Kossa staining. Thereby, the phosphate-bound calcium ions were replaced by silver ions visible as brown precipitates. Scale bar: 50 µm.
